# The surveillance of plague among rodents and dogs in Western Iran

**DOI:** 10.1371/journal.pntd.0011722

**Published:** 2023-11-10

**Authors:** Saber Esmaeili, Ahmad Mahmoudi, Parisa Esmaeili, Zohreh Yousefi Ghalejoogh, Alireza Mordadi, Ahmad Ghasemi, Ali Mohammadi, Amin Bagheri, Aria Sohrabi, Mina Latifian, Minoarisoa Rajerison, Javier Pizarro-Cerda, Ehsan Mostafavi

**Affiliations:** 1 National Reference Laboratory for Plague, Tularemia and Q Fever, Research Centre for Emerging and Reemerging Infectious Diseases, Pasteur Institute of Iran, Akanlu, Kabudar-Ahang, Hamadan, Iran; 2 Department of Epidemiology and Biostatics, Research Centre for Emerging and Reemerging Infectious Diseases, Pasteur Institute of Iran, Tehran, Iran; 3 Department of Biology, Faculty of Science, Urmia University, Iran; 4 Department of Microbiology, Research Center of Reference Health Laboratories, Ministry of Health and Medical Education, Tehran, Iran; 5 Department of Medical Entomology and Vector Control, School of Public Health and National Institute of Health Research, Tehran University of Medical Sciences, Tehran, Iran; 6 Plague Unit, Central Laboratory for Plague, Institute Pasteur de Madagascar, Antananarivo, Madagascar; 7 Yersinia Research Unit, Institute Pasteur, Paris, France; University of Texas Medical Branch at Galveston, UNITED STATES

## Abstract

**Background:**

The causative agent of plague, *Yersinia pestis*, is maintained in nature via a flea-rodent cycle. Western Iran is an old focus for plague, and recent data indicate that rodents and dogs in this region have serological evidence of *Y*. *pestis* infection. The purpose of this study was to conduct a large-scale investigation of *Y*. *pestis* infection in shepherd dogs, rodents, and their fleas in old foci for plague in Western Iran.

**Materials and methods:**

This study was conducted in Hamadan province from 2014 to 2020. Rodents and fleas were collected from various locations throughout this region. *Y*. *pestis* was investigated in rodent spleen samples and fleas using culture, serology, and real-time PCR methods. Additionally, sera samples were collected from carnivores and hares in this region, and the IgG antibody against the *Y*. *pestis* F1 antigen was assessed using an ELISA.

**Results:**

In this study, 927 rodents were captured, with *Meriones* spp. (91.8%) and *Microtus qazvinensis* (2.6%) being the most prevalent. A total of 6051 fleas were collected from rodents and carnivores, most of which were isolated from *Meriones persicus*. None of the rodents or fleas examined tested positive for *Y*. *pestis* using real-time PCR and culture methods. Meanwhile, IgG antibodies were detected in 0.32% of rodents. All serologically positive rodents belonged to *M*. *persicus*. Furthermore, none of the sera from the 138 carnivores (129 sheepdogs, five *Vulpes vulpes*, four *Canis aureus*), and nine hares tested positive in the ELISA test.

**Conclusion:**

This primary survey of rodent reservoirs shows serological evidence of *Y*. *pestis* infection. Western Iran is an endemic plague focus, and as such, it requires ongoing surveillance.

## Author summary

This article investigates the ongoing presence of the plague in Iran and stresses the importance of continuous monitoring in the western region, a major area for endemic plague. Rodents are the primary carriers of diseases in their natural habitats and contribute significantly to the ongoing presence of infectious cycles. The study focuses on the presence of *Yersinia pestis*, the bacteria responsible for the plague, in guard dogs, rodents, and fleas in the historical center of western Iran. The research confirms the presence of *Y*. *pestis* infection in rodents, specifically Meriones persicus, in western Iran through serological evidence. Given the new discoveries in Iran and the resurgence of the plague in neighboring countries, ongoing monitoring of plague infections in wildlife across various regions of the country is crucial for public health outcomes.

## Introduction

Plague is a highly contagious and potentially fatal disease caused by the gram-negative bacterium *Yersinia pestis* [1]. Plague is both an endemic disease and a potential biological weapon. Due to the availability of effective antibiotics, the mortality rate from endemic plague remains low worldwide. Despite significant advancements in sanitation, hygiene and clinical treatment, the plague continues to occur in some regions, with epidemics occurring annually [2]. In recent decades, human plague cases have resurfaced in several countries (e.g., Algeria, Libya, Jordan, Saudi Arabia, Afghanistan, and India) as a reemerging disease [3, 4]. Iran is the Middle East’s most important plague focus [5,6], with nine outbreaks (156 deaths) between 1947 and 1966 [7]. Although no human cases have been reported in Iran since, several outbreaks in neighboring countries (e.g., Kazakhstan in 1992 [8], Saudi Arabia in 1994 [9], Jordan in 1997 [10], and Afghanistan in 2007 [11] have been reported, as well as recent evidence of *Y*. *pestis* circulation in wildlife in western Iran [6], highlighting the entire region as a high-risk area for plague infection.

Currently, 279 rodent species are known to be infected by *Y*. *pestis*, of which only >25% are resistant or susceptible species taking part critical role in plague maintenance in wildlife and they are different from one region to another [12]. Throughout previous studies conducted in western Iran, jirds of the genus *Meriones* have been identified as the primary reservoirs [13,14]. While *M*. *persicus* and *M*. *libycus* are relatively resistant to *Y*. *pestis* infection, *M*. *tristrami* and *M*. *vinogradovi* are susceptible [15,16].

The current study conducted serologic, culture, and molecular tests on trapped mammals and fleas to determine the presence of plague infection in western Iran.

## Materials and methods

### Ethics statement

The ethical considerations of the study were approved by the Institutional Animal and Human Ethical Committee under national and international standards with a code of IR.PII.REC.1395.9, and the protocol was endorsed by the Pasteur Institute of Iran.

### Study area

Animals were trapped from a historic plague focus (600 km^2^) between 2014 and 2020. The area is located in eastern Kurdistan Province, on the northern border of Hamadan Province and the southwestern border of Zanjan Province, and is home to nearly 20,000 residents.

### Rodent sampling

The wooden life handmade traps, measuring 25×15×15 cm3, were utilized to capture rodents. Traps baited with dates and cucumber were set in the early evening, and the captured animals were transported to a nearby research laboratory the following morning. Geographic coordinates for sampling were recorded using the Global Positioning System (GPS). The rodents captured were identified using morphological characteristics and taxonomic keys [17,18].

Fleas were isolated from captured rodents by brushing the hairs of trapped animals into the collection sites’ water. Taxonomic keys were used to identify the fleas collected [19,20]. Fleas were used for culture and molecular analysis following their identification.

Blood was drawn from the rodent’s cardiac puncture. Centrifuged blood samples were isolated and stored at -20°C. Additionally, spleen samples were dissected and stored at 4°C under sterile conditions. All sera and spleen samples obtained were microbiologically analyzed.

### Carnivore sampling

After obtaining the consent of their owners, blood samples were collected from sheepdogs in the studied region. Hunting was used for capturing wild carnivores and lagomorphs, and blood samples were taken. Sera were isolated and stored at -20°C after blood samples were centrifuged.

### Culture

Rodent spleen and flea samples were homogenized in normal saline using a laboratory porcelain mortar and pestle in Class II plus biological safety cabinet, and 50 μl of the suspension was cultured on Yersinia Selective Agar (CIN) and MacConkey agar mediums and incubated at 28°C for 72 hours (checked daily). Gram staining was used to identify the suspected colonies, and biochemical tests including catalase, oxidase, lactose fermentation, citrate, urease activity, indole and motility were performed.

### Serology

All serum samples were tested using an enzyme-linked immunosorbent assay (ELISA) to determine the presence of antibodies against the *Y*. *pestis* Fraction 1 (F1) antigen [21]. The Institut Pasteur de Madagascar provided all materials required for the ELISA test.

### DNA extraction and Real-time PCR

Genomic DNA was extracted from rodent spleens and fleas using commercial kits (QIAamp DNA Mini Kit (Qiagen, Germany) and Viragene Tissue DNA Extraction (Viragene, Iran), respectively, according to the manufacturer’s instructions. All extracted DNA was stored at -20°C until molecular testing.

The Rotor-Gene 6600 Real-time PCR system (Corbett life science) was used to perform the real-time PCR [22]. Primers and probes for the yihN (chromosomal), *caf1* (pMT1 plasmid), and *pla* (pPCP1 plasmid) genes were used in the PCR (Table 1). The amplification program was as follows: Initial denaturation at 95°C for 10 minutes, followed by 45 cycles of 15 seconds at 95°C and 60 seconds at 58°C.

**Table 1:** Primers and probes used to detect *Yersinia pestis* in this study.

Target	Primer/Probe	Sequences (5’ to 3’)	Amplicon Size (bp)
*yihN* (chromosome)	Forward Reverse Probe	CGCTTTACCTTCACCAAACTGAAC GGTTGCTGGGAACCAAAGAAGA Cy5-TAAGTACATCAATCACACCGCGACCCGCTT-BHQ2	128
*caf1* (pMT1)	Forward Reverse Probe	CCGTTATCGCCATTGCATTATTTGG GCCAAGAGTAAGCGTACCAACAAG Hex-AAGCACCACTGCAACGGCAACTCTT-BHQ1	194
*pla* (pPCP1)	Forward Reverse Probe	ATTGGACTTGCAGGCCAGTATC ATAACGTGAGCCGGATGTCTTC FAM-AAATTCAGCGACTGGGTTCGGGCACA-BHQ1	144

## Results

**Fig 1:**
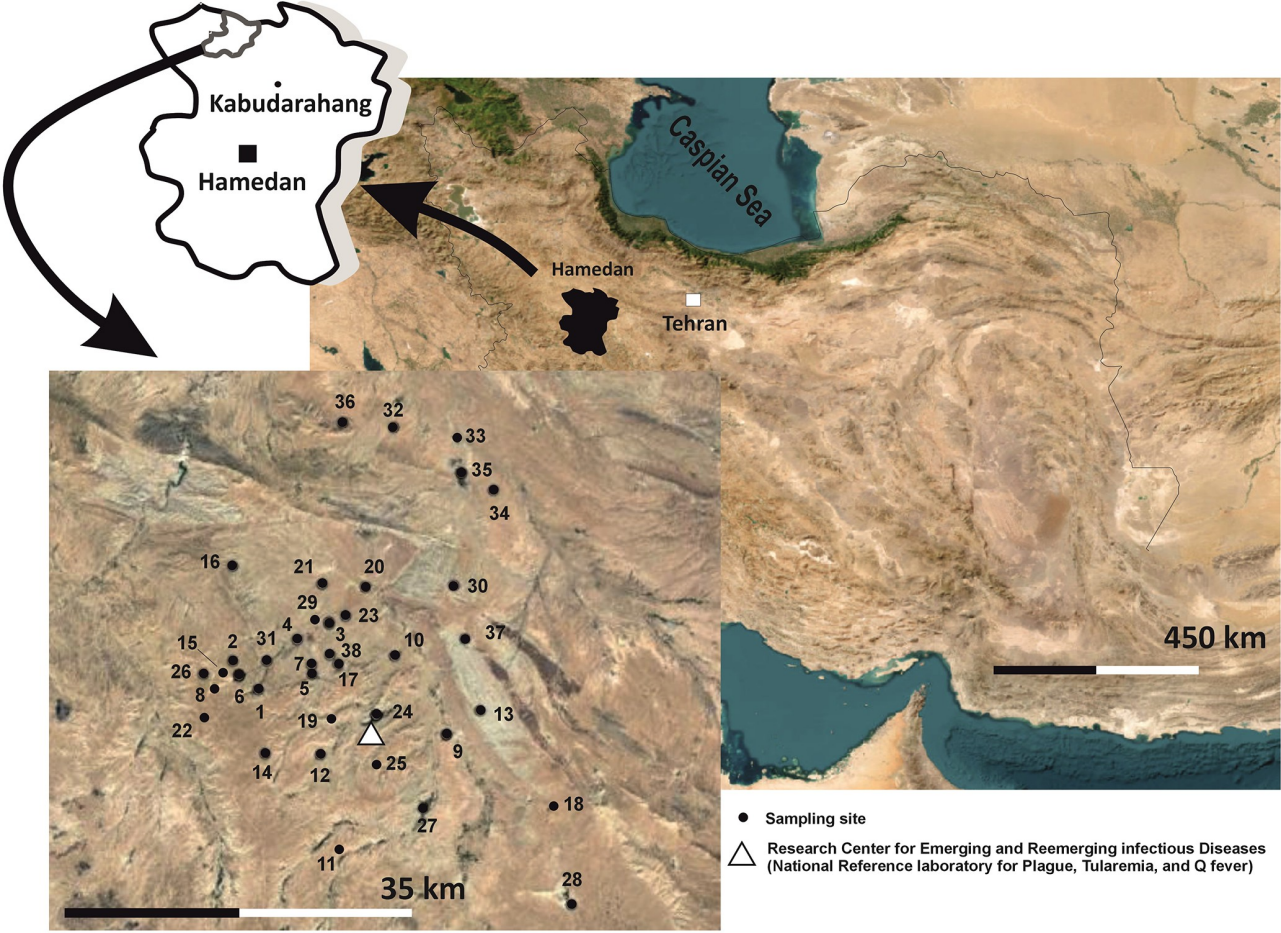
Map of the study region in Iran. A) The geographic position of Hamedan Province, the surveyed district map is shown in left hand, B) Sampling sites for rodents, and carnivores within historical plague focus. (1. Kohneh Hesar 2. Qeytar Mezruk 3. Bikaseh 4. Qazanqareh 5. Bashghurtaran 6. Qamish Dareh 7. Yekeh Guney 8. Och Darreh 9. Haji-Abad Darasi 10. Sorkhab 11. Tazeh Kand 12. Pir Badam 13. Yekeh Chalab Klik 14. Klik 15. Ban Quyu Darreh 16. Chopoqly 17. Aq Dash 18. Ab-Meshkin 19. Akanlu 20. Darreh Sinaf 21. Akhar Picheh 22. Reza Darasi 23. Shahgudar 24. Dash Quyu Darreh 25. Dagh Dali 26. Aq Bolagh Morshed 27. Masjed Olya 28. Shirin Su 29. Kohe bikase 30. Sarin bolagh 31. Su Darasi 32. Tokmeh tash 33. Ban Tokmeh tash 34. Aq bolagh torbaghi 35. Yekeh darreh 36. Agh bolaghe kaka abbasi 37. Agh bolaghe Reza Darasi 38. Bien Agh dash-Bashqortaran). For both parts (A & B), we used QGIS software and the map layers were obtained from Natural Earth: Maps: https://www.naturalearthdata.com, all layers and maps are public domain (http://www.naturalearthdata.com/about/terms-of-use/), and do not need permission for publication. The presented clip-arts are drawn by authors using a graphic design software (CorelDraw). We did not use any resource or base layer which need permission.

Between 2014 and 2020, 39 locations were investigated for this study. The area is surrounded by historical plague foci (Fig 1). Laboratory testing was performed on 927 rodents, 138 carnivores (129 sheepdogs, five *Vulpes vulpes*, four *Canis aureus*), and nine hares (*Lepus europaeus*).

Among the captured rodents, the dominant rodents were jirds of the genus *Meriones* (n = 851, 91.8%). *M*. *persicus* accounted for 84.49% (n = 719), *M*. *libycus* accounted for 8.93% (n = 76), *M*. *vinogradovi* accounted for 5.4% (n = 46), and *M*. *tristrami* accounted for 1.18% (n = 10) of the collected jirds. Additionally, 2.6% of the captured rodents (n = 24) belonged to *Microtus qazvinensis*. The remaining species comprised a small percentage of the captured rodents, including *Ellobius lutescens* (n = 15, 1.62%), *Spermophilus fulvus* (n = 6, 0.65%), *Arvicola persicus* (n = 6, 0.65%), *Nothocricetulus migratorius* (n = 4, 0.43%), *Mus musculus* (n = 4, 0.43%), *Calomyscus elburzensis* (n = 3, 0.32%), *Scarturus indicus* (n = 3, 0.32%), *S*. *williamsi* (n = 3, 0.32%), *Apodemus witherbyi* (n = 3, 0.32%), *Mesocricetus brandti* (n = 3, 0.32%), and *M*. *macedonicus* (n = 2, 0.22%), (for detailed information see Table 2).

**Table 2:** Sampling locations, and species names of the collected mammals and their fleas in the present study, during 2014–2020.

Studied region	Species of trapped rodent (N)
Ban Kohneh Hesar	*Meriones persicus* (41), *Meriones vinogradovi* (13), *Ellobius lutescens* (10), *Microtus qazvinensis* (2), *Calomyscus elburzensis* (2), *S*. *williamsi* (3)
Qeytar Mezruk	*M*. *persicus* (27), *Microtus qazvinensis* (3)
Bikaseh	*M*. *persicus* (29), *M*. *qazvinensis* (1)
Qazanqareh	*M*. *persicus* (113), *Meriones libycus* (42), *M*. *vinogradovi* (4), *Microtus qazvinensis* (2), *Mesocricetus brandti* (1)
Bashghurtaran	*M*. *persicus* (250), *M*. *libycus* (9), *Meriones tristrami* (1), *Ellobius lutescens* (1), *Apodemus witherbyi* (2), *M*. *qazvinensis* (1)
Qamish Dareh	*M*. *persicus* (2)
Yekeh Guney	*M*. *persicus* (10), *M*. *qazvinensis* (1)
Och Darreh	*M*. *persicus* (17), *M*. *tristrami* (1)
Haji-Abad Darasi	*M*. *persicus* (3), *M*. *libycus* (2), *M*. *vinogradovi* (5)
Sorkhab	*M*. *persicus* (2), *M*. *libycus* (1), *M*. *vinogradovi* (4), *Scarturus indicus* (3)
Tazeh Kand	*M*. *persicus* (2), *M*. *libycus* (2), *M*. *vinogradovi* (2), *M*. *tristrami* (1), *M*. *qazvinensis* (1)
Pir Badam	*M*. *persicus* (18), *M*. *tristrami* (4), *M*. *qazvinensis* (3), *Nothocricetulus migratorius* (2), *E*. *lutescens* (2)
Yekeh Chalab Klik	*M*. *persicus* (10), *M*. *libycus* (1), *M*. *qazvinensis* (1), *M*. *tristrami* (3)
Klik	*M*. *persicus* (1), *M*. *vinogradovi* (2), *M*. *brandti* (1)
Ban Quyu Darreh	*M*. *persicus* (1), *M*. *vinogradovi* (3)
Chopoqly	*M*. *vinogradovi* (3), *M*. *qazvinensis* (1)
Aq Dash	M. *persicus* (42), *M*. *libycus* (6), *M*. *vinogradovi* (3)
Ab–Meshkin	*M*. *persicus* (17), *M*. *libycus* (7), *M*. *vinogradovi* (2)
Akanlu	*M*. *persicus* (15), *E*. *lutescens* (2), *Mus musculus* (4), *A*. *witherbyi* (1), *Arvicola persicus* (6)
Darreh Sinaf	*M*. *persicus* (18)
Akhar Picheh	*M*. *persicus* (19), *M*. *vinogradovi* (1)
Reza Darasi	*M*. *persicus* (10)
Shahgudar	*M*. *persicus* (2), *M*. *libycus* (6)
Dash Quyu Darreh	*M*. *persicus* (5), *M*. *qazvinensis* (1)
Daq Dali	*M*. *persicus* (7), *M*. *qazvinensis* (4), *N*. *migratorius (*2), *Mus macedonicus* (2)
Aq bolagh Morshed	*M*. *persicus* (1), *M*. *vinogradovi* (1), *M*. *brandti* (1)
Masjed Olya	*M*. *persicus (12)*, *C*. *elburzensis* (1)
Shirin Su	*Spermophilus fulvus* (6)
Kohe bikase	*M*. *persicus* (*7)*
Sarin bolagh	*M*. *persicus* (16)
Su Darasi	*M*. *persicus* (11)
Tokmeh tash	*M*. *persicus* (10)
Ban Tokmeh tash	*M*. *persicus* (5), *M*. *vinogradovi* (2)
Aq bolagh torbaghi	*M*. *persicus* (2), *M*. *qazvinensis* (3)
Yekeh darreh	*M*. *persicus* (5)
Agh bolaghe kaka abbasi	*M*. *vinogradovi* (1)
Agh bolaghe Reza Darasi	*M*. *persicus* (3)
Bien Agh dash—Bashqortaran	*M*. *persicus* (8)

A total of 6051 fleas were collected from rodents and carnivores in this study, most of which were isolated from *Meriones persicus*. *Xenopsylla buxtoni* (96.74%), *Xenopsylla nuttalli* (0.8%), *Xenopsylla astia* (0.59%), *Nosopsyllus medus* (1.07%), *Nosopsyllus iranus* (0.39%), and *Rhadinopsylla ucrainica* (0.39%) were the most prevalent fleas identified. Additionally, *Stenoponia tripectinata* (n = 6) was collected from a *Vulpes vulpes* and a *Canis aureus*.

Serologically positive samples were obtained from the Ban Kohneh-Hesar region through ELISA. Out of the rodent sera tested, 0.32% were positive for IgG antibodies against the *Y*. *pestis* F1 antigen, specifically belonging to *M*. *persicus* (Table 3). However, none of the sera samples from dogs, *V*. *vulpes*, and *C*. *aureus* tested positive for IgG antibodies against the *Y*. *pestis* F1 antigen. *Y*. *pestis* was not isolated from rodent spleen and flea samples cultured in the laboratory. *Y*. *pestis* was not detected in rodent spleen and flea samples using Real-time PCR. In rodents’ spleen samples, Real-time PCR assays using the *yihN* and *pla* genes revealed no infection with *Y*. *pestis*. However, molecular analysis of the *caf1* gene revealed three positive spleen samples which all belonged to *M*. *persicus*.

**Table 3:** Captured rodents and its seroprevalence rate for plague in this study in western Iran in 2014–2020.

Genus	Rodent species	No. of seropositive animals (%)
*Meriones*	*M*. *persicus*	3 (0.41)
	*M*. *libycus*	0 (0)
	*M*. *vinogradovi*	0 (0)
	*M*. *tristrami*	0 (0)
*Mus*	*M*. *musculus*	0 (0)
	*M*. *macedonicus*	0 (0)
*Microtus*	*M*. *qazvinensis*	0 (0)
*Ellobius*	*E*. *lutescens*	0 (0)
*Scarturus*	*S*. *indicus*	0 (0)
	*S*. *williamsi*	0 (0)
*Apodemus*	*A*. *witherbyi*	0 (0)
*Calomyscus*	*C*. *elburzensis*	0 (0)
*Nothocricetulus*	*N*. *migratorius*	0 (0)
*Arvicola*	*A*. *persicus*	0 (0)
*Spermophilus*	*S*. *fulvus*	0 (0)
*Mesocricetus*	*M*. *brandti*	0 (0)
**Total**		**3 (0.32)**

## Discussion

This study was conducted in historic plague foci in western Iran, and serological findings in rodents indicated that *Y*. *pestis* is still circulating in this region. Plague continues to be a global public health problem. Several plague epidemics have occurred in our studied region in the past. Despite the plague’s long history in Iran, it remains difficult to describe its history and current state in the country accurately. In recent years, countries in the Middle East and North Africa have reported plague outbreaks [23]. For decades, the plague may remain silent. Silent periods of plague in natural foci may last ten years or longer, following which human plague may re-emerge.

Recent outbreaks have also demonstrated that plague can resurface in areas after an extended absence. Algeria, for example, experienced a resurgence of plague after a half-century period with no confirmed human cases, resulting in challenging conditions in the country [24]. Similarly, India endured dire conditions following the 1994 plague outbreak, both in terms of hygiene and economics [25,26]. As a result, now is the time to intensify disease surveillance in high-risk areas where human cases have already been reported as epizootic and enzootic.

In Iran, natural foci of plague are located throughout the Western and Northwestern of the country, primarily in the west and northwestern regions, and alternate between active and dormant periods. These are critical areas for epidemiological research. Western Iran is still home to the natural foci of plague. Between 1947 and 1966, nine outbreaks of plague were reported, resulting in 156 deaths, mainly in Kurdistan province. The most recent report of a human plague from this historical region dates back to 1966 [23,27]. In a survey performed during 2011–2012, the seroprevalence of *Y*. *pestis* was reported to be 1.02% in rodents and 3.42% in sheepdogs [6]. It is expected that positive plague samples would be found in subsequent years, considering that no changes were made to the environment and no interventions were implemented after the 2011–2012 report. Given that the environment has remained unchanged since the previous report in 2011–2012, it is anticipated that all three genes (yihN, pla, and caf1) would be detected in the sample to validate the presence of *Y*. *pestis* bacteria. Further studies conducted in the Kurdistan province in 2013 revealed no positive cases in either 245 rodents or 153 fleas [28]. The current study establishes the infection’s continuous circulation in these historical foci.

Plague is believed to persist in enzootic cycles with partially resistant rodents for extended periods of low prevalence. Following infection with *Y*. *pestis*, the enzootic cycle has a low fatality rate. Several hypotheses exist for how *Y*. *pestis* survives during inter-epizootic periods including long-term persistence in hibernating hosts, fleas, or soil [29]. According to the concept of telluric, *Y*. *pestis* could survive in the burrows of dead rodents for several years and re-infect other rodents [30]. *M*. *persicus* was the most frequently captured rodent in this study. This rodent is relatively immune to infection with *Y*. *pestis*. Additionally, all seropositive samples included in our study were *M*. *persicus* (0.41%). In a previous study conducted in the same area (2011–2012), we observed a seropositive rodent in *M*. *persicus* [6]. Thus, the findings of this study corroborate previous research indicating that *Meriones* (particularly *M*. *persicus*) are the primary reservoirs of plague in Iran.

Our failure to detect the bacterium in the examined animals and fleas could be related to the low bacterial circulation in the study area which is normally anticipated during the quiescent phase of a given natural focus. It is important to note that during this time, the plague may spread in small microfoci among hosts with low intensity, making it challenging to detect. As well as, being small or hardly accessible, active microfoci might escape from notice by surveillance systems [12]. Another possible reason for the rarity of plague discovery in the subject could be interpreted under intensive alterations enforced by recent agricultural advances, and the improved lifestyle of the people in recent years. During the last few decades, there have been widespread changes in climate, cultural practices of people, lifestyle, and health care systems; these changes can all affect components of the plague cycle (host, vector, and pathogen) in different ways. However, the seropositive rodents provide conclusive evidence of the plague’s persistence in our study area. After infection with *Y*. *pestis*, IgG antibody is produced against the F1 antigen and persists in the body for several months, making this one of the most practical surveillance methods [31]. In this study, all rodent spleen and flea cultures were negative for *Y*. *pestis*. The chances of isolating *Y*. *pestis* from rodents and their fleas are extremely slim, as the culture must be positive for an active infection. Given that a small number of our samples had a history of previous exposure to *Y*. *pestis* (seropositive), the lack of bacterium isolation is reasonable. However, to obtain and isolate this bacterium in the future, it is recommended that a larger number of rodents and their flea’s specimens be evaluated to increase the likelihood of isolation.

To confirm *Y*. *pestis* infection, both the plasmid (pMT1 and pPCP1) and chromosomal genes of this bacterium must be present simultaneously [32]. According to our findings, *Y*. *pestis* was not detected in the spleen or fleas of rodents by multiplex Real-time PCR. Acquiring the pMT1 and pPCP1 plasmids is necessary to differentiate *Y*. *pestis* from *Y*. *pseudotuberculosis*. These plasmids are required for the plague infection cycle to occur. Both plasmids are plague-specific and had not previously been detected in any other bacteria. Plasminogen activator, encoded by the *pla* gene on plasmid pPCP1, is used to detect *Y*. *pestis* in humans and rodents according to plague diagnostic protocols due to the high abundance in each gene bacterium. Recent findings suggest that relying solely on the *pla* gene for detection may be deceptive, as the gene was recently identified in bacteria such as *Citrobacter koseri* and *E*. *coli*, calling into question the diagnostic basis for this gene [33].

Identifying all three genes (yihN, pla, and caf1) in the sample is crucial to confirm the presence of *Y*. *pestis* bacteria. In 2016, researchers reported the first genetically confirmed *Y*. *pestis* (*pla* gene) bacteria in Algeria, but they were later identified as *Citrobacter koseri* [34]. *C*. *koseri* and *E*. *coli* were also found in *Rattus rattus*, *R*. *norvegicus*, *Mus musculus*, and *Apodemus sylvaticus* from Canada and England in another paper published in the same year (2016), but they did not belong to *Y*. *pestis* [35]. A similar scenario occurred in the Netherlands to detect the *pla* gene in *R*. *rattus* and *R*. *norvegicus* [36]. In our study, we did not have any positive samples for the *pla* gene, but three rodents were positive for the *caf1* gene. Unfortunately, molecular investigation of rodent spleens in this study was conducted after culture studies and on frozen spleen samples, so further culture and follow-up of samples with a positive *caf1* gene were not possible. Based on the literature review, heretofore there have been no reports of the *caf1* gene or its homolog in other bacteria or organisms. This gene is located on the pMT1 plasmid, and this unique plasmid is considered to be specific for *Y*. *pestis*. The *caf1* gene encoded a capsule-like antigen, fraction 1 (F1), which is very critical for plague infection. The results obtained from our study might suggest the hypothesis of the transfer of the pMT1 plasmid to other bacteria, or the presence of the *caf1* gene homolog in other organisms. Nevertheless, there is a need for a very extensive study in this field to prove this hypothesis, and the current study presents very preliminary evidence in this regard.

## Conclusion

Overall, while the results of this study indicate the presence of *Y*. *pestis* in the studied animals, the absence of human or rodent cases in natural foci does not always imply the absence of plague and given the disease’s ability to wreak havoc on health and economy, continued surveillance of potential reservoirs and vectors is critical to preparing for the possibility of epidemic resurgence.

People living in contaminated areas and their surroundings should be interviewed to evaluate their understanding of plague disease in both humans and animals, while also informing local authorities such as the governor’s office and regional and local health networks about the spread of plague among rodents or carnivores within the area.
